# Running Velocity as a Methodological Factor in Footwear Testing: Fixed Versus Self-Selected Conditions

**DOI:** 10.3390/bioengineering13070751

**Published:** 2026-06-26

**Authors:** Pierre Kiesewetter, Thomas L. Milani, Christian Mitschke

**Affiliations:** 1 Research Group for Biomechanics and Sensory Function, Institute of Human Movement Science and Health, Chemnitz University of Technology, 09126 Chemnitz, Germany; thomas.milani@hsw.tu-chemnitz.de; 2 Olympic Training Center Saxony, 09125 Chemnitz, Germany; christian.mitschke@osp-sachsen.de

**Keywords:** biomechanics, footwear, inertial measurement unit, running

## Abstract

Running velocity is an important methodological factor in footwear research, given its potential influence on biomechanical parameters used to evaluate shoe-specific effects. Since most previous studies have assessed footwear effects at standardized running velocities, it remains unclear whether shoe-specific biomechanical response patterns differ when runners transition to individually selected faster outdoor running velocities. Therefore, this study examined whether footwear-related biomechanical responses were modified by running velocity. Twenty-two runners completed outdoor running trials at two running velocities in seven running shoes differing in cushioning and midsole bending stiffness. Four commonly used inertial measurement unit-derived biomechanical parameters were assessed to determine whether shoe-specific responses differed between the two velocity conditions. Across all parameters, no significant shoe condition × running velocity interaction effects were observed. Higher running velocities increased the magnitude of the measured parameters, while the tested interaction terms did not provide statistical evidence that the relative shoe-related response patterns were systematically altered within the investigated velocity range. These findings suggest that individually preferred running velocities may be suitable for comparing relative shoe effects in field-based footwear testing for the investigated parameters and velocity range.

## 1. Introduction

Running is one of the most widely practiced sports worldwide, both in recreational and competitive settings. Driven by its sustained popularity, a wide range of footwear technologies has been developed in recent years—from variations in midsole cushioning [1,2,3,4,5,6], midsole structure [7,8,9], and midsole stack height [10,11,12,13,14,15] to increased midsole bending stiffness (MBS) [16,17,18,19] via carbon plates. These features are generally intended to enhance performance and may also influence the biomechanical factors associated with injury risk [20,21]. The effects of these technologies have been quantified using biomechanical and physiological outcomes [22,23]. Depending on the research question, midsole cushioning properties, MBS, and shoe geometry have been manipulated to evaluate their effects on biomechanical and physiological outcomes. Frequently examined biomechanical parameters include peak tibial acceleration (PTA) as an indicator of impact-related loading [24,25,26], foot strike angle (FSA) as a descriptor of strike pattern [27,28,29], peak eversion velocity (evVel) as a marker of frontal-plane rearfoot motion [30,31,32], and peak angular velocity in the sagittal plane (PAV) as a proxy for plantarflexion velocity [27,33]. In addition, several studies have assessed physiological outcomes such as oxygen uptake and running economy [34,35,36,37,38].

Methodological choices, particularly running velocity, can substantially influence a study’s outcomes and should therefore be considered explicitly when evaluating footwear-specific effects [39,40,41]. Running velocity is especially relevant in this context because it affects various biomechanical parameters commonly used to assess footwear effects, including impact-related, strike-related, and kinematic measures [26,28]. This consideration is particularly pertinent because standardized test velocities in footwear studies span a broad range, from moderate to performance-oriented velocities [34,36,42,43,44]. Consequently, the biomechanical response to a given shoe may depend not only on its mechanical properties but also on the running velocity at which it is tested. This raises the methodological question of whether shoe-specific biomechanical effects observed at one test velocity can be generalized to other, more habitual running velocities.

Previous research has examined footwear effects from both biomechanical and performance-oriented perspectives. Biomechanical studies often use moderate running velocities (10–12 km/h) that better reflect recreational running [33,45], whereas performance-oriented studies, particularly on carbon-plated footwear, typically employ higher running velocities (>14 km/h) [36,42]. Across both lines of research, footwear-related effects may depend on running velocity.

Most existing research has been conducted under laboratory conditions, typically on treadmills. Although these settings allow for controlled testing, they may not fully capture habitual overground biomechanics. A systematic review showed that treadmill and outdoor running can differ in relation to several key parameters [46]. Outdoor measurements therefore provide a complementary and ecologically relevant perspective rather than a simple replacement for laboratory-based testing. The increasing use of inertial measurement unit (IMU)-based running gait analysis offers a practical approach for assessing running biomechanics in both laboratory and real-world settings [47]. Despite this potential, running velocity and footwear properties have only rarely been examined in combination, even though initial evidence suggests that footwear effects may vary with running velocity [18,40,41,48,49,50]. However, findings have been inconsistent and largely restricted to selected biomechanical parameters, rather than indicating a robust and generalizable footwear × running velocity interaction pattern across outcomes [13,18,40,41,48,49,51]. Moreover, previous studies have typically investigated selected footwear properties at fixed running velocities under laboratory conditions. It therefore remains unclear whether the relative direction and magnitude of shoe-specific effects remain consistent when runners transition from a standardized velocity to an individually preferred faster outdoor running velocity.

To address this gap, the present study examined whether footwear-related biomechanical responses differ between a standardized running velocity and an individually preferred faster training velocity. This question is methodologically relevant because footwear-related biomechanical studies commonly standardize running velocity to enhance experimental control, whereas their findings are often extrapolated to habitual running conditions. Outdoor testing was therefore chosen to examine this issue under controlled field-based conditions. Although this approach may capture selected aspects of outdoor running, it should be distinguished from unrestricted real-world running.

Four biomechanical parameters representing impact loading, strike pattern, and foot motion were analyzed: (I) PTA, (II) FSA, (III) evVel, and (IV) PAV. These parameters were assessed across seven running shoes with systematically varied mechanical characteristics, including cushioning and MBS. They were selected because they capture the biomechanical features commonly discussed in relation to both footwear properties and running velocity.

Based on previous findings, it was hypothesized that running velocity would affect the absolute magnitude of the investigated biomechanical parameters. However, given the inconsistent and outcome-specific evidence on shoe condition × running velocity interactions, no consistent interaction pattern was expected. Instead, footwear effects were expected to vary in direction and relative magnitude across biomechanical outcomes.

By combining standardized and individualized running velocities with IMU-based outdoor measurements, this study aims to provide a controlled field-based methodological approach for footwear testing. The findings are intended to improve the interpretation of footwear × running velocity interactions and to inform methodological decisions in applied footwear research.

## 2. Materials and Methods

### 2.1. Participants

In summary, 22 recreational rearfoot runners without lower-extremity injuries in the last six months participated in this study (5 women, 17 men). Participants’ characteristics were as follows: age: 29.0 ± 9.4 years; body height: 176.1 ± 5.5 cm; body mass: 71.0 ± 7.3 kg; weekly running distance: 19.7 ± 24.6 km/week; running experience: 10.5 ± 6.9 years; and shoe size: UK 8. The participants were given information about the purpose and design of the study and then signed an informed consent document and completed a questionnaire on demographic, anthropometric, and training-related information. All procedures were performed in accordance with the recommendations of the Declaration of Helsinki. This study was approved by the Ethics Committee of the Faculty of Behavioural and Social Sciences of the Chemnitz University of Technology (101620265).

### 2.2. Footwear Conditions and Mechanical Characterization

**Figure 1:**
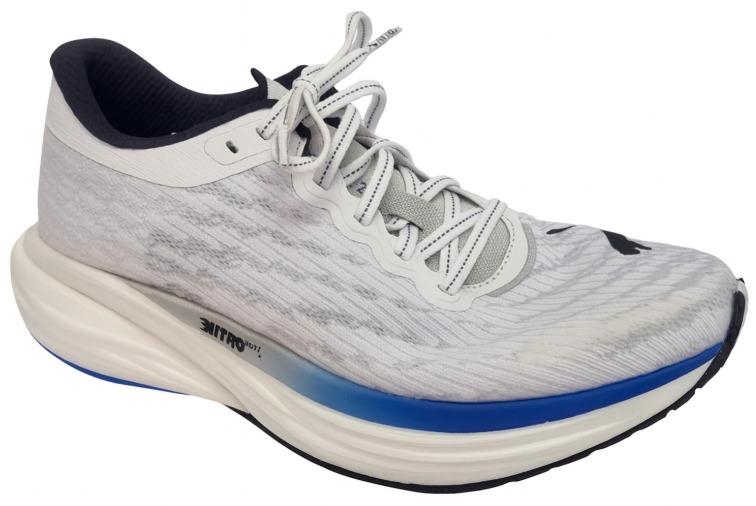
Test Shoe—PUMA Deviate Elite 2.

The PUMA Deviate Elite 2 (men’s UK size 8) was provided by PUMA and served as the reference model (S1) for this study, as it provided a suitable platform for systematically modifying midsole cushioning and MBS (Figure 1). The cushioning properties and MBS of the reference shoe were initially mechanically characterized by the research team. Based on these results, target specifications were defined to create conditions with softer or harder cushioning and more flexible or stiffer bending characteristics.

**Figure 2:**
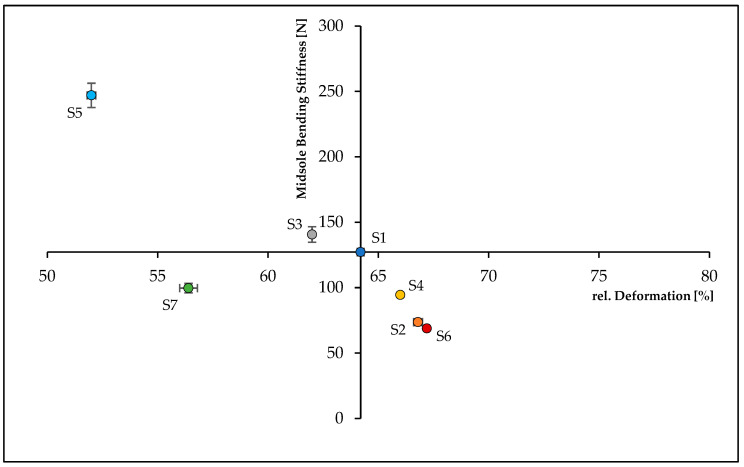
Overview of the mechanical properties of all shoes used in this study.

Six additional footwear conditions (S2–S7) were then manufactured by PUMA according to these study-specific target specifications (Figure 2). These modifications were implemented by altering the midsole materials and including or excluding a carbon plate while maintaining a consistent outsole, insole, and upper construction across all models. Externally, all shoes appeared identical. This design enabled a controlled investigation of the effects of cushioning and MBS on biomechanical outcomes.

Due to construction-related differences, primarily the amount of cushioning material and the presence or absence of a carbon plate, shoe mass varied slightly between footwear conditions (Table 1). To compensate for these differences, weighted boxes filled with lead material were attached to the heel region of each shoe. The heaviest model also received boxes without additional lead material to ensure that all shoes appeared externally identical. After the weights had been attached, the mass of each individual shoe was approximately 321 g.

Prior to mechanical testing and the main biomechanical assessment, all shoes were worn by five participants on a treadmill over a cumulative distance of 20 km per shoe to simulate moderate wear and induce material fatigue. The five participants involved in the standardized shoe-aging procedure were also part of the main study cohort. This standardized aging protocol was used to reduce variability in biomechanical responses due to initial stiffness or progressive material changes. It was intended to minimize differences in shoe condition between the first and last participants in the main study [52].

After the aging procedure, midsole stiffness and bending stiffness were quantified for each shoe using a servo-hydraulic testing system (HC10, Zwick GmbH & Co. KG, Ulm, Germany). Each shoe was tested five times following established protocols described by Schwanitz and Odenwald [53] and Kiesewetter et al. [23] (Table 1).

### 2.3. Experimental Setup and Data Collection

**Figure 3:**
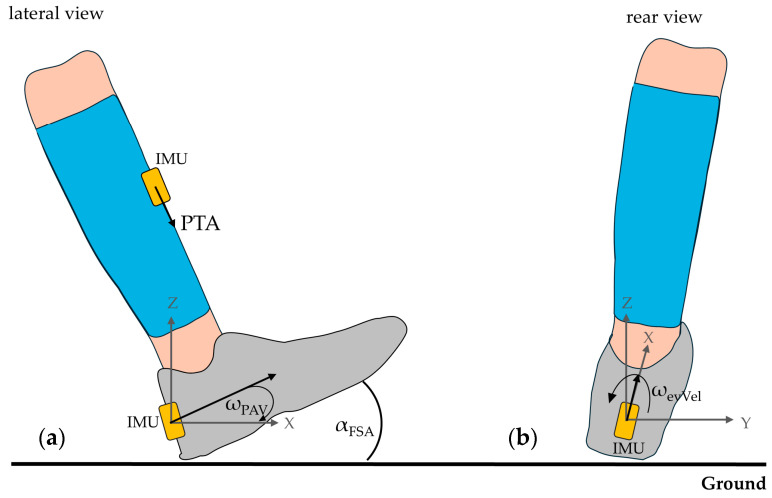
Sensor setup in lateral (**a**) and rear view (**b**) to measure biomechanical parameters’ peak tibial acceleration (PTA), foot strike angle (FSA), peak angular velocity (PAV) and peak eversion velocity (evVel). The calf compression sleeves are shown in blue and the IMUs in yellow.

Biomechanical data were collected during a 500 m run on a flat concrete sidewalk outside the laboratory using IMUs (Opal, APDM Inc., Portland, OR, USA; mass: 25 g), which integrated a tri-axial accelerometer (range: ±1982 m/s^2^) and a tri-axial gyroscope (range: ±2000°/s). Data were sampled at 800 Hz. According to Kiesewetter et al. [23], one IMU was affixed to the shaved medial aspect of the right tibia, midway between the malleolus and the tibial plateau, using double-sided adhesive tape (Figure 3a). To minimize movement artifacts caused by the sensor’s mass, the IMU was additionally secured with an elastic strap and a calf compression sleeve. The sensitive axis of the IMU was aligned with the longitudinal axis of the tibia, following the procedure described by Hennig et al. [24]. A second IMU was mounted on the heel counter of the right running shoe using a rigid, lightweight sensor holder fabricated via three-dimensional (3D) printing and secured with additional elastic tape (Figure 3b).

The experimental protocol consisted of two test blocks corresponding to the two running velocities. In block 1, participants ran at a fixed, moderate running velocity (v1) of 11 km/h (3.0 m/s)—selected as a representative moderate running velocity frequently used in recreational running research and as a common reference condition for cross-shoe comparisons [33,43,44,45]. In block 2, they ran at an individually selected brisk continuous running velocity (v2) (13.5 ± 1.3 km/h) intended to reflect habitual outdoor training pace. The order of the two velocity blocks was fixed by design and was not randomized. The standardized 11 km/h condition was completed first to minimize the likelihood that measurements in this condition would be affected by residual fatigue from preceding faster trials. In contrast, the shoe order was randomized across participants and separately within each velocity block. To monitor running velocity, a test supervisor accompanied each participant on a bicycle equipped with a calibrated speedometer [33]. Inclusion was restricted to habitual rearfoot strikers (RFS) to reduce biomechanical heterogeneity, as foot strike patterns differ in terms of impact transients, loading rates, and joint mechanics, and footwear properties may interact with strike pattern [54]. RFS was visually confirmed during the warm-up. After that, each participant completed a 500 m run in one pair of test shoes at the fixed running velocity (block 1). No separate shoe-specific familiarization period was included before the recorded trials. Breaks were provided during shoe changes, and subsequent trials were started once participants reported feeling sufficiently recovered. Outdoor testing was conducted under dry weather conditions, with ambient temperatures of approximately 17–22 °C and no extreme weather conditions such as rain, strong wind, or excessive heat. This procedure was repeated until all seven footwear conditions had been tested at that running velocity. The entire protocol was then repeated at the self-selected velocity (block 2). Within each block, running velocity was held constant across shoe conditions.

### 2.4. Data Analysis

Data from the sensors were analyzed post hoc using MATLAB R2024a (MathWorks™, Natick, MA, USA). Prior to analysis, signal noise was reduced by applying a fourth-order, zero-phase Butterworth low-pass filter with cutoff frequencies of 80 Hz for accelerometer data and 50 Hz for gyroscope data [23]. Stride segmentation within the continuous dataset was performed using the vertical acceleration signal from the IMU attached to the heel counter, which was processed with a zero-phase Butterworth high-pass filter (cutoff: 80 Hz). The first peak in the filtered signal was defined as the initial ground contact (IC) of the foot [55].

PTA was identified as the maximum acceleration measured by the tibia-mounted IMU within 200 ms after IC. FSA at IC was calculated as the shoe orientation angle in the sagittal plane based on data from the heel-mounted IMU, according to the method described by Mitschke et al. [56]. EvVel was determined by analyzing the maximum angular velocity in the frontal plane of the shoe-mounted sensor, following Mitschke et al. [30]. Foot rollover was assessed based on PAV in the sagittal plane within 200 ms after IC, in line with the approach of Bräuer et al. [33].

To eliminate acceleration and deceleration artifacts at the beginning and end of each trial, the first and last ten steps of each dataset were excluded from the analysis. For each remaining stride, one value was calculated for each outcome (PTA, FSA, evVel, and PAV). These stride-level values were subsequently averaged within each participant, shoe condition, and velocity condition.

### 2.5. Statistical Analysis

All statistical analyses were performed using IBM SPSS Statistics (Version 31.0; IBM Corp., Armonk, NY, USA). The resulting participant-level condition means were used as input for the statistical models. For each outcome variable (PTA, FSA, evVel, and PAV), a separate linear mixed-effects model was fitted to account for the repeated measures design. Shoe condition (seven levels), running velocity, and their interaction were included as fixed effects, with participants included as a random intercept to account for repeated observations within individuals. Running velocity was modeled as a continuous predictor in the primary analysis because the self-selected faster velocity varied between participants and therefore did not represent a single fixed velocity level. This approach allowed the actual running velocity of each trial to be considered and enabled the estimation of velocity-related changes per 1 km/h.

As a sensitivity analysis, all models were repeated with running velocity treated as a categorical factor representing the standardized and self-selected faster velocity conditions. The interaction term was evaluated first, and main effects were interpreted only in the absence of a significant shoe condition × running velocity interaction. Formal equivalence or non-inferiority testing was not performed because no predefined and biomechanically justified equivalence margins were available for the investigated IMU-derived outcomes and shoe-ranking comparisons. To descriptively quantify rank-order consistency between velocity conditions, Spearman rank correlations were calculated between shoe-specific mean values at the standardized and self-selected faster velocity for each outcome and reported in the Appendix A (Table A1).

For significant omnibus main effects of shoe condition, Bonferroni-adjusted pairwise post hoc comparisons of estimated marginal means were performed. Shoe-specific velocity slopes were estimated as the change in the outcome per 1 km/h and reported with 95% confidence intervals in the Appendix A (Table A2). In addition, interaction estimates were extracted from the fixed-effect parameter estimates and reported with 95% confidence intervals in the Appendix A (Table A3). These estimates represent differences in velocity-related changes between each shoe condition and the reference shoe S1 and describe the range of shoe-specific interaction effects compatible with the observed data. A post hoc sensitivity power analysis was conducted using G*Power (Version 3.1.9.7; Heinrich Heine University Düsseldorf, Düsseldorf, Germany) to estimate the smallest detectable interaction effect size. The analysis was specified as a repeated measures within-subject design with one group, 14 repeated measurements, a total sample size of 22 participants, α = 0.05, and 80% power. Statistical significance was set at α = 0.05 for all analyses. Effect sizes for fixed effects were reported as partial eta squared (η_p_^2^), with values of 0.01, 0.06, and 0.14 interpreted as small, medium, and large effects, respectively [57].

## 3. Results

**Figure 4:**
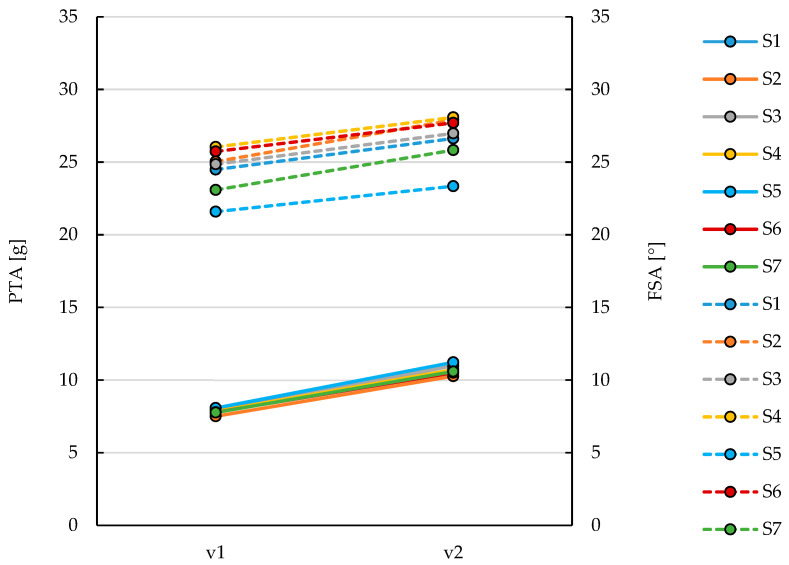
Mean values of peak tibial acceleration (PTA; solid lines) and foot strike angle (FSA; dashed lines) across shoe conditions for both running velocities v1 and v2.

**Figure 5:**
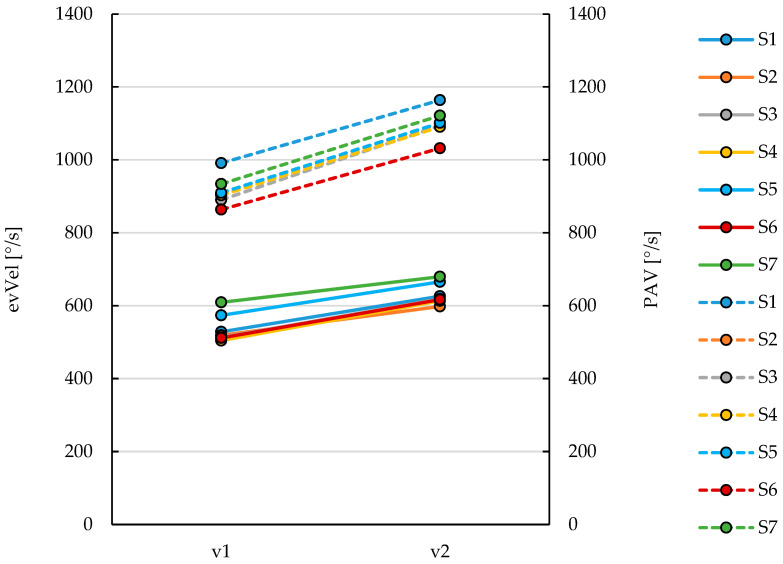
Mean values of peak eversion velocity (evVel; solid lines) and peak angular velocity in sagittal plane (PAV; dashed lines) across shoe conditions for both running velocities v1 and v2.

Across shoe conditions, an average of 229.5 ± 10.4 steps was included for the standardized velocity (v1 = 11 km/h). At the self-selected velocity, 198.4 ± 18.3 steps were included (v2 = 13.5 ± 1.3 km/h; range: 12–16 km/h). The descriptive statistics for all biomechanical parameters are presented in Table 2 and illustrated in Figure 4 and Figure 5. Model outputs from the linear mixed-effects analysis (F, *p*, η_p_^2^) are summarized in Table 3.

### 3.1. Peak Tibial Acceleration (PTA)

For PTA, the mean values at v1 ranged from 7.5 ± 2.1 g (S2) to 8.1 ± 2.4 g (S5), and at v2 from 10.3 ± 3.4 g (S2) to 11.2 ± 3.4 g (S5) (Figure 4, Table 2). Significant main effects were present for shoe condition (*p* = 0.005; η_p_^2^ = 0.07) and running velocity (*p* < 0.001; η_p_^2^ = 0.77) (Table 3).

### 3.2. Foot Strike Angle (FSA)

At v1, mean FSA was lowest in S5 (21.6 ± 4.5°) and highest in S4 (26.0 ± 4.3°), whereas at v2 values ranged from 23.3 ± 5.0° (S5) to 28.1 ± 4.6° (S4) (Figure 4, Table 2). Shoe condition and running velocity both affected FSA (shoe condition: *p* < 0.001; η_p_^2^ = 0.66; running velocity: *p* < 0.001; η_p_^2^ = 0.59) (Table 3).

### 3.3. Peak Eversion Velocity (evVel)

For evVel, mean values at v1 ranged from 504.3 ± 116.9°/s (S4) to 609.2 ± 135.6°/s (S7), and at v2 from 598.0 ± 171.9°/s (S2) to 679.6 ± 166.7°/s (S7) (Figure 5, Table 2). The analysis showed the effects of shoe condition (*p* < 0.001; η_p_^2^ = 0.30) and running velocity (*p* < 0.001; η_p_^2^ = 0.56) (Table 3).

### 3.4. Peak Angular Velocity (PAV)

At v1, the mean values for PAV ranged from 863.6 ± 99.3°/s (S6) to 991.1 ± 126.3°/s (S1), and at v2 from 1031.9 ± 114.7°/s (S6) to 1163.3 ± 143.0°/s (S1) (Figure 5, Table 2). For PAV, effects were observed for shoe condition (*p* < 0.001; η_p_^2^ = 0.46) and running velocity (*p* < 0.001; η_p_^2^ = 0.87) (Table 3).

Additional analyses are provided in the Appendix A. Shoe-specific slopes of the velocity-related changes were positive across all four biomechanical outcomes and increased with running velocity across shoe conditions (Table A2, Figure 4 and Figure 5). Spearman rank correlations showed moderate-to-high rank-order consistency between velocity conditions for PTA (ρ = 0.929, *p* = 0.003), FSA (ρ = 0.964, *p* < 0.001), evVel (ρ = 0.786, *p* = 0.036), and PAV (ρ = 0.964, *p* < 0.001) (Table A1). Interaction estimates with 95% confidence intervals are presented in Table A3. Sensitivity analyses with running velocity treated as a categorical factor yielded the same overall interpretation as the primary continuous-velocity models. No significant shoe condition × running velocity interaction was observed for PTA (*p* = 0.948), FSA (*p* = 0.344), evVel (*p* = 0.678), or PAV (*p* = 0.856) (Table A4). The post hoc sensitivity power analysis indicated that the present design was sensitive to effects of approximately f = 0.174 or larger, corresponding to η_p_^2^ = 0.029, with 80% power at α = 0.05.

## 4. Discussion

This study investigated whether shoe-related differences in selected biomechanical running parameters were modified when runners transitioned from a standardized running velocity to an individually preferred, higher training velocity. The main finding was that running velocity increased the absolute magnitude of the investigated biomechanical parameters, whereas the statistical models did not indicate a significant shoe condition × running velocity interaction for any of the four parameters (Table 3). Within the tested running velocity range, the present findings provide no clear statistical evidence that shoe-related biomechanical differences were systematically altered by running velocity. This interpretation was also supported by the categorical sensitivity analysis, which yielded the same overall conclusion as the primary continuous-velocity models (Table A4).

The supplementary analyses helped to further characterize this finding. Shoe-specific velocity slopes were positive across all outcomes (Table A2), indicating that running velocity primarily affected the absolute magnitude of the measured parameters. Spearman rank correlations suggested moderate-to-high descriptive rank-order similarity between velocity conditions (Table A1), while the interaction estimates and their 95% confidence intervals mostly included zero (Table A3). However, these findings should not be interpreted as evidence of equivalence, complete stability, or true invariance between velocity conditions. In addition, the post hoc sensitivity analysis indicated that smaller shoe condition × running velocity interaction effects may have remained undetected.

This pattern is methodologically relevant because shoe construction is known to influence running biomechanics, while standardized test protocols and the generalizability of findings to different running conditions remain unresolved issues in the literature [58]. Furthermore, the use of IMU-based outdoor measurements addresses a limitation of purely laboratory-based approaches, particularly because overground and treadmill running are not entirely biomechanically equivalent [47,59,60]. The present findings are also consistent with the general observation that running velocity influences lower extremity kinematics and kinetics, even across relatively narrow running velocity ranges [61]. In contrast, shoe-related effects are often parameter-specific and depend on the manipulated design feature [58].

One possible explanation is that runners may adjust to shoe condition and running velocity in a way that limits pronounced changes in the relative shoe-related response patterns. From this perspective, increasing running velocity could generally intensify biomechanical demands, whereas the relative influence of shoe characteristics was not clearly shown to differ within the tested velocity range. Nevertheless, interaction effects are fundamentally possible. For example, Yang et al. [5] reported running velocity-dependent effects of midsole stiffness on lower extremity joint angles and plantar loading across several relative running velocity levels. Therefore, while shoe × running velocity interactions may exist, the present statistical models did not provide evidence for such interactions for PTA, FSA, evVel, and PAV within the running velocity range tested here.

### 4.1. PTA

PTA is particularly relevant because it is commonly used as an indicator of impact-related loading during running [25,26]. Against this background, the PTA findings can be interpreted in light of the overall pattern of results.

No significant shoe × running velocity interaction was observed for PTA. Thus, the present model did not provide statistical evidence that shoe-related PTA differences were systematically modified by running velocity within the tested velocity range. A similar overall pattern was reported by Lam et al. [51]. They found significant effects of running velocity on tibial shock and impact loading, as well as shoe-related differences between various cushioning conditions. Their findings also showed that running speed substantially altered the absolute load level. More generally, reviews of PTA measurements indicate that running velocity is one of the strongest influencing factors on PTA during running [62].

The main effect of velocity observed in the present study is therefore in good agreement with previous work. Furthermore, the main effect of shoe condition suggests that the tested shoes differed in their impact-related mechanical behavior, which is consistent with findings showing that different shoe categories can alter the characteristics of tibial shock [63]. However, the interpretation of PTA should be approached with caution: recent studies have questioned the extent to which PTA can be considered a direct surrogate parameter for tibial bone loading. Accordingly, the present PTA findings should be discussed as evidence for impact-related external loading characteristics rather than as a direct estimate of injury risk.

Overall, the PTA results support the study’s general conclusion: both running velocity and shoe condition influenced the magnitude of the variable, while the model did not provide evidence for a systematic velocity-dependent modification of the shoe-related PTA differences.

### 4.2. FSA

FSA is an established kinematic measure used to describe the running-style-specific foot strike patterns in running and is defined by the orientation of the foot at the moment of IC. Its inclusion in the evaluation of running shoes is therefore methodologically relevant [64].

In line with the findings for PTA, no significant shoe × running velocity interaction was observed for FSA. This indicates that FSA was influenced by shoe condition and running velocity, but that the model did not indicate a systematic velocity-dependent shift in shoe-related FSA differences. This pattern is consistent with Fredericks et al. [40], who reported that shoe condition and running speed independently and significantly influenced ankle angle at ground contact. It is also broadly consistent with Lai et al. [65], who found that the distribution of foot strike pattern was more strongly influenced by foot condition (shod vs. barefoot) than by running velocity, with shoed runners tending to maintain a heel strike pattern across different velocities.

The observed main effect of running speed on FSA is plausible considering the previous literature, although the direction of speed-related changes is not always consistent. For example, Forrester and Townend [28] showed that FSA responses were heterogeneous between runners across running velocities of 2.2 to 6.1 m/s, suggesting that higher velocities can be achieved via different coordinative solutions. This could explain why some studies report greater dorsiflexion at contact with increasing running velocity, while others describe a shift toward greater plantarflexion in velocity-dependent foot strike parameters.

A strong main effect of shoe condition on FSA was also observed in the present study, supporting previous findings that shoe characteristics such as midsole geometry, cushioning, and sole construction can influence foot strike mechanics [12,58]. Although Wang et al. [66] reported running velocity-dependent effects on foot strike during barefoot versus shod running, this comparison involved a larger difference between conditions than the shoes tested here. This may explain why shoe-related differences were observed but did not vary systematically with running velocity.

### 4.3. EvVel

EvVel describes the rate of rearfoot motion in the frontal plane shortly after IC and is a relevant parameter of foot pronation during running [67]. Elevated evVel values have been discussed in relation to medial tibial stress syndrome and other overuse-related complaints [68]. Therefore, evVel and its time of occurrence are commonly used in running shoe research to assess the effects of shoe condition [69,70] and running velocity [30] on rearfoot mechanics.

No significant shoe × running velocity interaction effect was observed for evVel. The available data therefore did not provide clear statistical evidence that shoe-related differences in frontal-plane rearfoot motion depended systematically on whether runners ran at the standardized or the faster, self-selected running velocity. This pattern is closely consistent with the findings of Meinert et al. [31], who reported significant main effects of shoe configuration and running velocity on maximum pronation velocity, but no interaction between the two factors.

The significant main effect of running velocity found here is also consistent with previous work showing that maximum pronation velocity or evVel increases with increasing running velocity [31]. The main effect of shoe condition is also supported by existing literature. For example, MacLean et al. [70] reported lower maximum rearfoot eversion velocities in harder than in softer shoes, indicating that material properties of the midsole can alter rearfoot dynamics in the frontal plane.

Taken together, the available evVel findings suggest that rearfoot motion in the frontal plane increased at higher velocities and differs between shoes, while the model did not provide clear evidence for a systematic interaction between shoe condition and running velocity.

### 4.4. PAV

PAV is defined as the maximum angular velocity of the shoe in the sagittal plane after IC and thus characterizes the downward rotation of the shoe during the heel-to-forefoot transition [33]. It serves as an objective measure of the functional rollover behavior (“ride”) of the running shoe and reflects a central aspect of perceived running comfort, which can be influenced by fatigue [71].

No significant shoe × running velocity interaction was found for PAV. The literature on interaction effects for this specific variable is limited, but previous studies suggest that sagittal roll-off dimensions are sensitive to shoe construction and can also change with velocity-dependent distal foot mechanics. In particular, Bräuer et al. [33] showed under outdoor field conditions that PAV differed between shoes that differed only in MBS. Furthermore, PAV was strongly related to the subjective perception of ride. Kiesewetter et al. [23] also reported significant differences in PAV between shoes during a 10 km treadmill run in carbon-plated shoes.

The pronounced main effect of running velocity observed in the present study is biomechanically plausible, since a higher running velocity should accelerate the heel to forefoot transition. However, caution is advised when directly linking this to the literature, as interaction effects have been reported for other distal foot parameters. For example, Liu et al. [50] showed that running velocity and MBS interacted for the plantarflexion angle and the peak plantarflexion velocity of the metatarsophalangeal joint. This again supports the view that shoe × running velocity interactions are possible but highly parameter-specific. In the present study, however, the PAV model did not indicate that shoe-related differences in sagittal-plane angular velocity were systematically modified by running velocity within the tested range.

Although several main effects of shoe condition were statistically significant, the absolute differences between the shoes remained small in some cases. This was particularly evident for PTA, where the differences between the shoes were smaller than the velocity-related increase from v1 to v2. Therefore, statistically significant shoe effects should not automatically be interpreted as practical or clinically relevant.

From a methodological perspective, the present findings suggest that individually preferred running velocities could be considered for comparing relative shoe effects, at least within the tested running velocity range and for the four IMU-derived parameters investigated here. This is relevant because biomechanical shoe studies are often based on fixed running velocities for practical and experimental reasons, even though runners typically use shoes under more individualized running conditions. The present approach should therefore be interpreted as a controlled field-based testing protocol rather than as unrestricted real-world running. It may approximate selected aspects of overground running more closely than purely laboratory-based protocols while still allowing for controlled comparisons between the shoe conditions.

These findings should be interpreted within the specific context of the present study. The absence of significant interaction effects does not mean that running velocity is irrelevant for shoe research. Rather, it suggests that, for the participants tested here and within this running velocity range, running velocity changed the absolute level of the parameters more than the relative differentiation between the shoes. However, interaction effects could still occur at more extreme running velocities, with greater shoe variations, or with other biomechanical outcomes, as studies on midsole stiffness and distal foot mechanics have shown [5,18,48,50].

This study has several limitations. First, the generalizability of the present findings is limited by the heterogeneous composition of the sample in terms of performance level and training background, as well as by the absence of elite runners. This heterogeneity is reflected in the large variability in weekly running distance (19.7 ± 24.6 km/week), indicating substantial differences in habitual training volume among participants. Such variability may have contributed to interindividual differences in biomechanical responses. Consequently, transferability to high-performance populations remains restricted, and stratified analyses across performance levels were only feasible to a limited extent. Future studies should therefore include adequately powered and more homogeneous recreational, sub-elite and elite cohorts, where feasible.

Second, the experimental footwear conditions were not intended to represent the full range of commercially available running shoes. They were designed to systematically vary cushioning properties and midsole bending stiffness under controlled conditions based on a commercially available reference shoe. Therefore, the findings should be interpreted primarily in relation to these mechanical properties rather than as direct evidence for specific commercial shoe models or broader market categories.

Third, data were collected at a standardized running velocity (11 km/h) and at an individually preferred running velocity (mean: 13.5 km/h; range: 12–16 km/h). The latter showed substantial interindividual variability and may have introduced between-subject differences in relative exercise intensity and physiological load. A more rigorous approach would have been to individualize workload using objective pre-test performance diagnostics. Examples include cardiopulmonary exercise testing or lactate threshold testing. Exercise intensity could have been prescribed relative to individual physiological thresholds, such as first and second lactate thresholds (LT1 and LT2). This would likely have improved comparability across participants and reduced potential confounding due to differences in fitness, running economy, and physiological strain.

Fourth, although the present study was conducted outdoors, the protocol remained experimentally controlled and should not be considered equivalent to unrestricted habitual training. Only approximately 500 m were analyzed per shoe and velocity condition on a flat concrete surface with externally monitored running velocity. This distance may be insufficient to obtain stable within-subject estimates of IMU-derived biomechanical parameters. In addition, the velocity blocks were completed in a fixed order, meaning that between-velocity differences cannot be attributed exclusively to running velocity. Although this order was chosen to reduce fatigue-related carry-over from faster trials to the standardized 11 km/h condition, block-order effects, cumulative fatigue, and adaption effects related to repeated trials may still have influenced the recorded differences. Longer measurement distances or a defined acclimatization phase followed by the analysis of a steady-state section may yield more robust estimates. However, longer trials would have increased the total running distance per test day to approximately 14 km because seven shoe conditions and two velocities were tested, thereby increasing the risk of cumulative fatigue.

## 5. Conclusions

Within the investigated velocity range, running velocity increased the absolute magnitude of the measured IMU-derived biomechanical parameters, whereas the statistical models did not provide clear evidence that shoe-related response patterns were systematically modified by running velocity. This finding should be interpreted cautiously and within the context of the tested shoes, participants, velocity range, and biomechanical outcomes. Importantly, the absence of statistically significant shoe condition × running velocity interactions should not be interpreted as evidence of equivalence, true invariance, or proof that standardized running velocities can generally be replaced. Given the limited sensitivity of the present design, potentially relevant shoe condition × running velocity interaction effects cannot be ruled out. From an applied perspective, self-selected running velocities may therefore be considered as a possible option in controlled field-based footwear testing. However, this approach should not be interpreted as equivalent to unrestricted real-world running, because both velocity conditions remained experimentally constrained by the predefined route, monitored running velocity, standardized footwear conditions, and short trial distance. Future studies should clarify under which conditions self-selected running velocities can be reliably used in footwear testing, particularly across broader footwear conditions, running velocities, and runner populations.

## Figures and Tables

**Table 1:** Characteristics of shoe conditions for men’s size UK 8 (mean ± standard deviation (SD) of five trials).

	Weight [g]	Rearfoot Height [mm]	Relative Deformation [%]	Midsole Bending Stiffness [N]
S1	268.5	26.6 ± 0.1	64.2 ± 0.1	127.2 ± 2.8
S2	245.8	27.2 ± 0.2	66.8 ± 0.2	73.6 ± 2.5
S3	283.3	28.6 ± 0.1	62.0 ± 0.0	140.6 ± 5.9
S4	261.6	27.7 ± 0.1	66.0 ± 0.0	94.4 ± 1.7
S5	308.1	29.6 ± 0.2	52.0 ± 0.2	247.0 ± 9.3
S6	238.7	27.1 ± 0.2	67.2 ± 0.1	68.8 ± 0.8
S7	271.2	28.1 ± 0.1	56.4 ± 0.4	99.6 ± 3.6

**Table 2:** Mean ± standard deviation of peak tibial acceleration (PTA), foot strike angle (FSA), peak eversion velocity (evVel), and peak angular velocity (PAV) for both running velocity v1 = 11 km/h and v2 = 13.5 km/h (range: 12–16 km/h).

Parameter	Running Velocity	S1	S2	S3	S4	S5	S6	S7
PTA [g]	v1 v2	8.0 ± 2.0 11.1 ± 3.2	7.5 ± 2.1 10.3 ± 3.4	7.8 ± 2.0 11.0 ± 3.3	7.8 ± 2.1 10.7 ± 3.2	8.1 ± 2.4 11.2 ± 3.4	7.8 ± 2.1 10.5 ± 3.2	7.8 ± 2.3 10.6 ± 3.4
FSA [°]	v1 v2	24.5 ± 4.2 26.6 ± 4.8	25.0 ± 4.4 27.9 ± 4.6	24.9 ± 4.1 27.0 ± 4.6	26.0 ± 4.3 28.1 ± 4.6	21.6 ± 4.5 23.3 ± 5.0	25.7 ± 3.8 27.7 ± 4.7	23.1 ± 4.4 25.8 ± 4.8
evVel [°/s]	v1 v2	528.4 ± 137.6 626.0 ± 182.9	518.7 ± 122.3 598.0 ± 171.9	513.0 ± 130.7 618.4 ± 186.5	504.3 ± 116.9 613.5 ± 173.1	573.7 ± 147.1 665.5 ± 186.7	511.6 ± 115.2 616.9 ± 172.0	609.2 ± 135.6 679.6 ± 166.7
PAV [°/s]	v1 v2	991.1 ± 126.3 1163.8 ± 143.0	908.3 ± 106.2 1100.0 ± 148.7	890.3 ± 108.1 1093.6 ± 157.0	902.9 ± 118.6 1090.6 ± 149.0	909.4 ± 100.5 1101.6 ± 118.9	863.6 ± 99.3 1031.9 ± 114.7	933.7 ± 121.7 1121.4 ± 144.1

**Table 3:** The effects (*p*-value, η_p_^2^) between shoe condition and running velocity on peak tibial acceleration (PTA), foot strike angle (FSA), peak eversion velocity (evVel), and peak angular velocity (PAV).

Parameter	Effect	F	*p*	η_p_^2^
PTA [g]	Shoe Condition Running Velocity Shoe Condition × Running Velocity	3.22 907.37 0.69	0.005 * <0.001 * 0.657	0.07 0.77 0.01
FSA [°]	Shoe Condition Running Velocity Shoe Condition × Running Velocity	88.85 386.20 0.97	<0.001 * <0.001 * 0.446	0.66 0.59 0.02
evVel [°/s]	Shoe Condition Running Velocity Shoe Condition × Running Velocity	19.26 344.97 1.22	<0.001 * <0.001 * 0.294	0.30 0.56 0.03
PAV [°/s]	Shoe Condition Running Velocity Shoe Condition × Running Velocity	38.33 1802.60 1.41	<0.001 * <0.001 * 0.210	0.46 0.87 0.03

**Note**: Significant *p*-values (*p* < 0.05) are indicated by an asterisk (*).

## Data Availability

The data presented in this study are available on request from the corresponding author.

## Abbreviations

The following abbreviations are used in this manuscript:

MBS	Midsole Bending Stiffness
PTA	Peak Tibial Acceleration
FSA	Foot Strike Angle
evVel	Peak Eversion Velocity
PAV	Peak Angular Velocity in the sagittal plane
IMU	Inertial Measurement Unit
3D	Three-dimensional
RFS	Rear Foot Strikers
IC	Initial Ground Contact
LT1	First Lactate Threshold
LT2	Second Lactate Threshold

## Appendix A

**Table A1:** Spearman rank correlations of shoe-specific mean values between v1 and v2.

Parameter	Spearman’s ρ	*p*
PTA	0.929	0.003
FSA	0.964	<0.001
evVel	0.786	0.036
PAV	0.964	<0.001

**Table A2:** Shoe-specific velocity slopes with 95% confidence intervals.

Parameter	Shoe	Slope per 1 km/h	95% CI
PTA	S1	1.195	[0.993, 1.397]
[g/(km/h)]	S2	1.125	[0.923, 1.327]
	S3	1.272	[1.070, 1.474]
	S4	1.112	[0.910, 1.314]
	S5	1.276	[1.074, 1.478]
	S6	1.103	[0.901, 1.305]
	S7	1.063	[0.861, 1.265]
FSA	S1	0.917	[0.679, 1.155]
[°/(km/h)]	S2	1.027	[0.789, 1.265]
	S3	0.797	[0.559, 1.035]
	S4	0.866	[0.628, 1.104]
	S5	0.782	[0.544, 1.020]
	S6	0.803	[0.565, 1.041]
	S7	1.083	[0.845, 1.321]
evVel	S1	40.111	[29.212, 51.010]
[(°/s)/(km/h)]	S2	33.801	[22.902, 44.700]
	S3	46.445	[35.546, 57.344]
	S4	43.929	[33.030, 54.828]
	S5	39.514	[28.615, 50.413]
	S6	39.952	[29.053, 50.851]
	S7	28.238	[17.339, 39.137]
PAV	S1	68.522	[59.628, 77.416]
[(°/s)/(km/h)]	S2	73.844	[64.950, 82.738]
	S3	81.315	[72.421, 90.209]
	S4	73.751	[64.857, 82.645]
	S5	74.277	[65.383, 83.171]
	S6	63.998	[55.104, 72.892]
	S7	71.704	[62.810, 80.598]

**Table A3:** Differences in velocity-related slopes between shoe conditions and reference shoe S1, with 95% confidence intervals and *p*-value.

Parameter	Velocity-Slope Difference	Estimate	95% CI	*p*
PTA	S2–S1	−0.070	[−0.354, 0.215]	0.631
[g/(km/h)]	S3–S1	0.077	[−0.207, 0.362]	0.594
	S4–S1	−0.083	[−0.367, 0.202]	0.567
	S5–S1	0.081	[−0.203, 0.366]	0.575
	S6–S1	−0.092	[−0.377, 0.192]	0.525
	S7–S1	−0.132	[−0.417, 0.152]	0.361
FSA	S2–S1	0.110	[−0.227, 0.446]	0.521
[°/(km/h)]	S3–S1	−0.120	[−0.456, 0.216]	0.484
	S4–S1	−0.051	[−0.387, 0.286]	0.768
	S5–S1	−0.135	[−0.471, 0.201]	0.430
	S6–S1	−0.114	[−0.450, 0.223]	0.506
	S7–S1	0.166	[−0.170, 0.502]	0.331
evVel	S2–S1	−6.310	[−21.720, 9.100]	0.421
[(°/s)/(km/h)]	S3–S1	6.334	[−9.077, 21.744]	0.419
	S4–S1	3.818	[−11.593, 19.228]	0.626
	S5–S1	−0.597	[−16.007, 14.814]	0.939
	S6–S1	−0.159	[−15.569, 15.251]	0.984
	S7–S1	−11.873	[−27.283, 3.537]	0.130
PAV	S2–S1	5.322	[−7.255, 17.898]	0.406
[(°/s)/(km/h)]	S3–S1	12.793	[0.217, 25.370]	0.046
	S4–S1	5.229	[−7.347, 17.805]	0.414
	S5–S1	5.755	[−6.822, 18.331]	0.368
	S6–S1	−4.524	[−17.101, 8.052]	0.479
	S7–S1	3.182	[−9.394, 15.758]	0.619

**Table A4:** Sensitivity analysis comparing continuous and categorical velocity specifications for shoe condition, running velocity, and their interaction effects.

Parameter	Model	Shoe *p*	Running Velocity *p*	Shoe × Running Velocity *p*
PTA	continuous	0.005	<0.001	0.657
	categorical	0.069	<0.001	0.948
FSA	continuous	<0.001	<0.001	0.446
	categorical	<0.001	<0.001	0.344
evVel	continuous	<0.001	<0.001	0.294
	categorical	<0.001	<0.001	0.678
PAV	continuous	<0.001	<0.001	0.210
	categorical	<0.001	<0.001	0.856

## References

[1] Sterzing T., Schweiger V., Ding R., Cheung J.T.M., Brauner T. (2013). Influence of Rearfoot and Forefoot Midsole Hardness on Biomechanical and Perception Variables during Heel-Toe Running. Footwear Sci..

[2] Sterzing T., Custoza G., Ding R., Cheung J.T.M. (2015). Segmented Midsole Hardness in the Midfoot to Forefoot Region of Running Shoes Alters Subjective Perception and Biomechanics during Heel-Toe Running Revealing Potential to Enhance Footwear. Footwear Sci..

[3] Yang Z., Cui C., Zhou Z., Zheng Z., Yan S., Liu H., Qu F., Zhang K. (2024). Effect of Midsole Hardness and Surface Type Cushioning on Landing Impact in Heel-Strike Runners. J. Biomech..

[4] Malisoux L., Urhausen A., Flores N., Theisen D., Morio C. (2024). Running Shoe Cushioning Properties at the Rearfoot and Forefoot and Their Relationship to Injury: Study Protocol for a Randomised Controlled Trial on Leisure-Time Runners. BMJ Open Sport Exerc. Med..

[5] Yang L., Liu X., Liu Y., Liu J., Yan S., Fei G. (2025). The Impact of Midsole Hardness on Joint Angles and Plantar Loading during Running at Multiple Running Velocities. Front. Public Health.

[6] Mitschke C., Karger K., Milani T.L. (2019). Differences in Mechanical Midsole Characteristics of Running Shoes Do Not Influence Physiological Variables in Aerobic and Anaerobic Running. J. Hum. Kinet..

[7] Knoepfli-Lenzin C., Waech J.C., Gülay T., Schellenberg F., Lorenzetti S. (2014). The Influence of a New Sole Geometry While Running. J. Sports Sci..

[8] Wunsch T., Alexander N., Kröll J., Stöggl T., Schwameder H. (2017). Effects of a Leaf Spring Structured Midsole on Joint Mechanics and Lower Limb Muscle Forces in Running. PLoS ONE.

[9] Ryu S., Stefanyshyn D., Kong S., Park S.K. (2021). Effects of a Curved Heel Shape in a Running Shoe on Biomechanical Variables and Comfort. Appl. Sci..

[10] Chambon N., Delattre N., Guéguen N., Berton E., Rao G. (2014). Is Midsole Thickness a Key Parameter for the Running Pattern?. Gait Posture.

[11] Law M.H.C., Choi E.M.F., Law S.H.Y., Chan S.S.C., Wong S.M.S., Ching E.C.K., Chan Z.Y.S., Zhang J.H., Lam G.W.K., Lau F.O.Y. (2019). Effects of Footwear Midsole Thickness on Running Biomechanics. J. Sports Sci..

[12] Zhang Z., Lake M. (2022). A Re-Examination of the Measurement of Foot Strike Mechanics During Running: The Immediate Effect of Footwear Midsole Thickness. Front. Sports Act. Living.

[13] Kettner C., Stetter B.J., Stein T. (2025). The Effects of Different Shoe Stack Heights and Running Speeds on Full-Body Running Coordination: An Uncontrolled Manifold Analysis. J. Biomech..

[14] Koegel J., Huerta S., Gambietz M., Ullrich M., Heyde C., Dorschky E., Eskofier B. (2024). Clustering Runners’ Response to Different Midsole Stack Heights: A Field Study. Sensors.

[15] Hannigan J.J., Pollard C.D. (2020). Differences in Running Biomechanics between a Maximal, Traditional, and Minimal Running Shoe. J. Sci. Med. Sport.

[16] Cigoja S., Firminger C.R., Asmussen M.J., Fletcher J.R., Edwards W.B., Nigg B.M. (2019). Does Increased Midsole Bending Stiffness of Sport Shoes Redistribute Lower Limb Joint Work during Running?. J. Sci. Med. Sport.

[17] Cigoja S., Asmussen M.J., Firminger C.R., Fletcher J.R., Edwards W.B., Nigg B.M. (2020). The Effects of Increased Midsole Bending Stiffness of Sport Shoes on Muscle-Tendon Unit Shortening and Shortening Velocity: A Randomised Crossover Trial in Recreational Male Runners. Sports Med. Open.

[18] Day E., Hahn M. (2020). Optimal Footwear Longitudinal Bending Stiffness to Improve Running Economy Is Speed Dependent. Footwear Sci..

[19] Roy J.P.R., Stefanyshyn D.J. (2006). Shoe Midsole Longitudinal Bending Stiffness and Running Economy, Joint Energy, and EMG. Med. Sci. Sports Exerc..

[20] Agresta C., Giacomazzi C., Harrast M., Zendler J. (2022). Running Injury Paradigms and Their Influence on Footwear Design Features and Runner Assessment Methods: A Focused Review to Advance Evidence-Based Practice for Running Medicine Clinicians. Front. Sports Act. Living.

[21] Kim H., Ahn J. (2025). Technologically Advanced Running Shoes Reduce Biomechanical Factors of Running Related Injury Risk. Sci. Rep..

[22] Nigg B.M., Nigg S., Hoitz F., Subramanium A., Vienneau J., Wannop J.W., Khassetarash A., Alizadeh S., Matijevich E., Honert E.C. (2023). Highlighting the Present State of Biomechanics in Shoe Research (2000–2023). Footwear Sci..

[23] Kiesewetter P., Bräuer S., Haase R., Nitzsche N., Mitschke C., Milani T.L. (2022). Do Carbon-Plated Running Shoes with Different Characteristics Influence Physiological and Biomechanical Variables during a 10 Km Treadmill Run?. Appl. Sci..

[24] Hennig E.M., Milani T.L., Lafortune M.A. (1993). Use of Ground Reaction Force Parameters in Predicting Peak Tibial Accelerations in Running. J. Appl. Biomech..

[25] Lafortune M.A. (1991). Three-Dimensional Acceleration of the Tibia during Walking and Running. J. Biomech..

[26] Sheerin K.R., Besier T.F., Reid D. (2020). The Influence of Running Velocity on Resultant Tibial Acceleration in Runners. Sports Biomech..

[27] Heidenfelder J., Sterzing T., Bullmann M., Milani T.L. (2008). Heel Strike Angle and Foot Angular Velocity in the Sagittal Plane during Running in Different Shoe Conditions. J. Foot Ankle Res..

[28] Forrester S.E., Townend J. (2015). The Effect of Running Velocity on Footstrike Angle—A Curve-Clustering Approach. Gait Posture.

[29] Cheung R., Wong R., Chung T., Choi R., Leung W., Shek D. (2016). Relationship between Foot Strike Pattern, Running Speed, and Footwear Condition in Recreational Distance Runners. Sports Biomech..

[30] Mitschke C., Öhmichen M., Milani T. (2017). A Single Gyroscope Can Be Used to Accurately Determine Peak Eversion Velocity during Locomotion at Different Speeds and in Various Shoes. Appl. Sci..

[31] Meinert I., Bichler S., Brown N., Alt W., Colloud F., Domalain M., Monnet T. (2015). Effects of Footwear and Running Speed on Foot Kinematics in the Frontal Plane. Proceedings of the 33rd International Conference on Biomechanics in Sports.

[32] Brindle R.A., Foch E., Smoliga J.M., Westbrook A.E., Hegedus E.J., Ford K.R. (2026). Changes in Biomechanical Risk Factors for Injury Across Normalized Running Speeds in Healthy Collegiate Cross-Country Runners. J. Appl. Biomech..

[33] Bräuer S., Kiesewetter P., Milani T.L., Mitschke C. (2021). The ‘Ride’ Feeling during Running under Field Conditions—Objectified with a Single Inertial Measurement Unit. Sensors.

[34] Dinato R.C., Cruz R., Azevedo R.A., Hasegawa J.S., Silva R.G., Ribeiro A.P., Lima-Silva A.E., Bertuzzi R. (2021). Footwear Designed to Enhance Energy Return Improves Running Economy Compared to a Minimalist Footwear: Does It Matter for Running Performance?. Braz. J. Med. Biol. Res..

[35] Hoogkamer W., Kipp S., Spiering B.A., Kram R. (2016). Altered Running Economy Directly Translates to Altered Distance-Running Performance. Med. Sci. Sports Exerc..

[36] Joubert D.P., Jones G.P. (2022). A Comparison of Running Economy across Seven Highly Cushioned Racing Shoes with Carbon-Fibre Plates. Footwear Sci..

[37] Worobets J., Wannop J.W., Tomaras E., Stefanyshyn D. (2014). Softer and More Resilient Running Shoe Cushioning Properties Enhance Running Economy. Footwear Sci..

[38] Hébert-Losier K., Finlayson S.J., Driller M.W., Dubois B., Esculier J.-F.F., Beaven C.M. (2022). Metabolic and Performance Responses of Male Runners Wearing 3 Types of Footwear: Nike Vaporfly 4%, Saucony Endorphin Racing Flats, and Their Own Shoes. J. Sport Health Sci..

[39] Brughelli M., Cronin J., Chaouachi A. (2011). Effects of Running Velocity on Running Kinetics and Kinematics. J. Strength Cond. Res..

[40] Fredericks W., Swank S., Teisberg M., Hampton B., Ridpath L., Hanna J.B. (2015). Lower Extremity Biomechanical Relationships with Different Speeds in Traditional, Minimalist, and Barefoot Footwear. J. Sports Sci. Med..

[41] Yu P., He Y., Gu Y., Liu Y., Xuan R., Fernandez J. (2022). Acute Effects of Heel-to-Toe Drop and Speed on Running Biomechanics and Strike Pattern in Male Recreational Runners: Application of Statistical Nonparametric Mapping in Lower Limb Biomechanics. Front. Bioeng. Biotechnol..

[42] Hoogkamer W., Kipp S., Frank J.H., Farina E.M., Luo G., Kram R., Beck O.N., Golyski P.R., Sawicki G.S., Aminaka N. (2018). A Comparison of the Energetic Cost of Running in Marathon Racing Shoes. Sports Med..

[43] Perl D.P., Daoud A.I., Lieberman D.E. (2012). Effects of Footwear and Strike Type on Running Economy. Med. Sci. Sports Exerc..

[44] Warne J., Moran K., Warrington G. (2018). Small Step Frequency Changes Due to Footwear Condition Have No Effect on Running Economy. Sports Med. Int. Open.

[45] Hashizume S., Murai A., Hobara H., Kobayashi Y., Tada M., Mochimaru M. (2017). Training Shoes Do Not Decrease the Negative Work of the Lower Extremity Joints. Int. J. Sports Med..

[46] Van Hooren B., Fuller J.T., Buckley J.D., Miller J.R., Sewell K., Rao G., Barton C., Bishop C., Willy R.W. (2020). Is Motorized Treadmill Running Biomechanically Comparable to Overground Running? A Systematic Review and Meta-Analysis of Cross-Over Studies. Sports Med..

[47] Benson L.C., Räisänen A.M., Clermont C.A., Ferber R. (2022). Is This the Real Life, or Is This Just Laboratory? A Scoping Review of IMU-Based Running Gait Analysis. Sensors.

[48] Day E.M., Hahn M.E. (2021). Does Running Speed Affect the Response of Joint Level Mechanics in Non-Rearfoot Strike Runners to Footwear of Varying Longitudinal Bending Stiffness?. Gait Posture.

[49] Trama R., Blache Y., Hautier C. (2019). Effect of Rocker Shoes and Running Speed on Lower Limb Mechanics and Soft Tissue Vibrations. J. Biomech..

[50] Liu Q., Chen H., Song Y., Alla N., Fekete G., Li J., Gu Y. (2022). Running Velocity and Longitudinal Bending Stiffness Influence the Asymmetry of Kinematic Variables of the Lower Limb Joints. Bioengineering.

[51] Lam W.K., Liebenberg J., Woo J., Park S.K., Yoon S.H., Cheung R.T.H., Ryu J. (2018). Do Running Speed and Shoe Cushioning Influence Impact Loading and Tibial Shock in Basketball Players?. PeerJ.

[52] Chambon N., Sevrez V., Ly Q.H., Guéguen N., Berton E., Rao G. (2014). Aging of Running Shoes and Its Effect on Mechanical and Biomechanical Variables: Implications for Runners. J. Sports Sci..

[53] Schwanitz S., Odenwald S. (2008). Long-Term Cushioning Properties of Running Shoes. The Engineering of Sport 7.

[54] Sun X., Yang Y., Wang L., Zhang X., Fu W. (2018). Do Strike Patterns or Shoe Conditions Have a Predominant Influence on Foot Loading?. J. Hum. Kinet..

[55] Mitschke C., Heß T., Milani T.L. (2017). Which Method Detects Foot Strike in Rearfoot and Forefoot Runners Accurately When Using an Inertial Measurement Unit?. Appl. Sci..

[56] Mitschke C., Zaumseil F., Milani T.L. (2017). The Influence of Inertial Sensor Sampling Frequency on the Accuracy of Measurement Parameters in Rearfoot Running. Comput. Methods Biomech. Biomed. Engin..

[57] Cohen J. (1988). Statistical Power Analysis for the Behavioral Sciences.

[58] Xiaole S., Wing-Kai L., Xini Z., Junqing W., Weijie F., Sun X., Lam W.K., Zhang X., Wang J., Fu W. (2020). Systematic Review of the Role of Footwear Constructions in Running Biomechanics: Implications for Running-Related Injury and Performance. J. Sports Sci. Med..

[59] Stankiewicz M., Saternus S., Błażkiewicz M., Kędziorek J., Cwyl K., Kłak-Dziemian A. (2025). Biomechanical Differences between Overground and Treadmill Running in Professional Runners—A Pilot Study. Acta Bioeng. Biomech..

[60] Hill M., Kiesewetter P., Milani T.L., Mitschke C. (2024). An Investigation of Running Kinematics with Recovered Anterior Cruciate Ligament Reconstruction on a Treadmill and In-Field Using Inertial Measurement Units: A Preliminary Study. Bioengineering.

[61] Orendurff M.S., Kobayashi T., Tulchin-Francis K., Tullock A.M.H., Villarosa C., Chan C., Kraus E., Strike S. (2018). A Little Bit Faster: Lower Extremity Joint Kinematics and Kinetics as Recreational Runners Achieve Faster Speeds. J. Biomech..

[62] Sheerin K.R., Reid D., Besier T.F. (2019). The Measurement of Tibial Acceleration in Runners—A Review of the Factors That Can Affect Tibial Acceleration during Running and Evidence-Based Guidelines for Its Use. Gait Posture.

[63] Xiang L., Gu Y., Rong M., Gao Z., Yang T., Wang A., Shim V., Fernandez J. (2022). Shock Acceleration and Attenuation during Running with Minimalist and Maximalist Shoes: A Time- and Frequency-Domain Analysis of Tibial Acceleration. Bioengineering.

[64] Altman A.R., Davis I.S. (2012). A Kinematic Method for Footstrike Pattern Detection in Barefoot and Shod Runners. Gait Posture.

[65] Lai Y.J., Chou W., Chu I.H., Wang Y.L., Lin Y.J., Tu S.J., Guo L.Y. (2020). Will the Foot Strike Pattern Change at Different Running Speeds with or without Wearing Shoes?. Int. J. Environ. Res. Public Health.

[66] Wang R., Fukuda D.H., Cheng P., Hu Y., Stout J.R., Hoffman J.R. (2020). Differential Effects of Speed on Two-Dimensional Foot Strike Pattern during Barefoot and Shod Running in Recreationally Active Men. Sports Biomech..

[67] Nigg B., Behling A.V., Hamill J. (2019). Foot Pronation. Footwear Sci..

[68] Jun H.P., Chang E. (2020). Rearfoot and Tibial Motion during Gait Associated with Medial Tibial Stress Syndrome: A Systematic Review. Exerc. Sci..

[69] Spencer M., Goldcamp N., McCrory J.L. (2025). Influence of Carbon-Fiber Shoes on Outdoor Running Biomechanics as Assessed with Wearable Sensors. Int. J. Exerc. Sci..

[70] MacLean C.L., Davis I.S., Hamill J. (2009). Influence of Running Shoe Midsole Composition and Custom Foot Orthotic Intervention on Lower Extremity Dynamics during Running. J. Appl. Biomech..

[71] Mitschke C., Heß T., Milani T.L., Kiesewetter P. (2025). Fatigue-Related Biomechanical Changes During a Half-Marathon Under Field Conditions Assessed Using Inertial Measurement Units. Biomechanics.

